# Computed Tomographic Evaluation of the Sacroiliac Joints of Young Working Labrador Retrievers of Various Work Status Groups: Detected Lesions Vary Among the Different Groups and Finite Element Analyses of the Static Pelvis Yields Repeatable Measures of Sacroiliac Ligament Joint Strain

**DOI:** 10.3389/fvets.2020.00528

**Published:** 2020-08-14

**Authors:** Michael Carnevale, Jeryl Jones, Gang Li, Julia Sharp, Katherine Olson, William Bridges

**Affiliations:** ^1^Department of Animal and Veterinary Sciences, Clemson University, Clemson, SC, United States; ^2^Department of Mechanical Engineering, Clemson University, Clemson, SC, United States; ^3^Department of Statistics, Colorado State University, Fort Collins, CO, United States; ^4^School of Mathematical and Statistical Sciences, Clemson University, Clemson, SC, United States

**Keywords:** computed tomography, sacroiliac joint, finite element analysis, finite element modeling, working dogs, sacroiliac joint disease

## Abstract

Musculoskeletal injuries can lead to a working dog being withdrawn from service prior to retirement. During training exercises, young working dogs are often required to perform repetitive tasks, including adoption of an upright posture (or “hupp” task). Non-invasive, quantitative methods would be helpful for supporting research on effects of these repetitive tasks on sacroiliac joints (SIJ). Furthering our understanding of lesions in and biomechanical stresses on the SIJ could provide insight into possible training modifications for minimizing risks of SIJ injury. Aims of this retrospective, secondary analysis, exploratory study were to test hypotheses that (1) mean numbers of SIJ computed tomographic (CT) lesions/dog would differ among work status groups in young working Labrador Retrievers; (2) a methodology for using CT data and finite element analysis (FEA) to quantify SIJ ligament strain in the static canine pelvis would be feasible; and (3) this FEA methodology would yield repeatable measures of SIJ ligament strain. Clinical and CT data for 22 Labrador retriever working dogs, aged 11–48 months, were retrospectively reviewed. Dogs were categorized into three work status groups (Breeder, Detection, Other). A veterinary radiologist who was unaware of dog group status recorded numbers of CT lesions for each SIJ, based on previously published criteria. Mean numbers of SIJ CT lesions/dog were compared among dog work status groups. An *a priori* FEA model was created from the CT images of one of the dogs using image analysis software packages. Using tissue properties previously published for the human pelvis, various directional loads (*n* = 8) and forces (48 ligament strain values) were placed on the canine model in five trials. Repeatability was tested using regression analysis. There was a significantly greater mean number of subchondral sclerosis lesions in left SIJ of Breeder vs. Detection dogs, a significantly greater mean number of subchondral cysts in right SIJ for Detection vs. Breeder dogs, and a significantly greater mean number of subchondral cysts in right SIJ of Other vs. Breeder dogs (*p* < 0.05). Finite element modeling and analysis using CT data was feasible and yielded repeatable results in 47/48 (98%) of tests at each combination of strain, ligament, and side.

## Introduction

Working dogs are important contributors for police, security, search and rescue, and military missions throughout the world (1–4). Detection training and working tasks often require working dogs to repetitively assume an upright stance (“hupp” position), with all of their weight placed on their hind legs and therefore with increased forces being applied to their sacroiliac joints (SIJ) (1). It has been proposed that, because SIJ are innervated with pain receptors, injury, or degenerative disease may be one of the causes of lower back pain in working dogs (1, 5). Rigorous training in repetitive agility-type motions in young dogs has also been proposed to increase the likelihood of developing chronic joint injuries later in life due to the under-developed physes (6). In people, this premise has been supported, given that most spinal injuries in young athletes have been found to occur after a sudden increase in the intensity and frequency of training (7–9). This clinical problem has been termed “overtraining” (10). Bone scintigraphy of the sacroiliac joint (SIJ) in young human athletes reporting lower back pain showed increased radiopharmaceutical uptake in one or both joints signifying increased bone turnover in athletes reporting lower back pain without any known specific trauma or reported radiographic abnormalities (7, 9). Cumulative musculoskeletal injuries are the most common injuries among working dogs and an important cause of early retirement (1, 3, 4). Published studies describing sacroiliac joint (SIJ) lesions in working dogs and methods for assessing possible effects of working tasks on canine SIJ are currently lacking.

The complex anatomy of canine SIJ has been previously detailed (11, 12). The SIJ consist of both synovial and fibrocartilaginous components. Joints are surrounded by the sheet-like dorsal and ventral sacroiliac ligaments. These ligaments play a role in stabilization of the SIJ and pelvis. The SIJ is also supported by interosseous ligaments connecting the articular surfaces of the sacral and ilial wings and a sacrotuberous ligament that connects the caudodorsal margins of the S3 vertebra to the dorsal margins of the ischiatic tuberosities of the ischium. The sacrotuberous ligament also plays a role in limiting pelvic range of motion. It has been proposed that hormonal changes in intact female dogs could predispose them to developing calcifications in the SIJ (5, 13). In puppies, the paired right and left hemispheres of the pelvis are joined together by a pelvic symphysis (11). In puppies, this is a fibrous ligament. As the dog ages, that ligament starts to ossify merging the two hemispheres into one. There is limited evidence evaluating the effects of strenuous exercise in dogs with open physes, however studies in rats report conflicting evidence that, while exercise may limit longitudinal bone growth and cause epiphyseal trabecular thinning in young animals, it may also lead to increased bone mineral density, cortical thickness and improved load bearing (14, 15).

Non-invasive techniques for quantifying SIJ lesions and theoretically quantifying stress and strain in sacroiliac joint ligaments would be helpful for supporting future research on effects of working and/or training tasks on the SIJ of young working dogs. Furthering our understanding of the presence of lesions in-, and biomechanical stresses on the SIJ could provide necessary insight into detection, effective screening, surveillance, and training modifications for mitigating conditions that increase the risk of SIJ injury and modification of training protocols for optimal performance and career longevity. Computed tomography (CT) has been validated as a method for characterizing SIJ lesions based on comparisons with gross pathology in a study of cadaver canine specimens (16). However, descriptions of CT SIJ lesions in working dogs have not been published. Mechanical stress and strain forces applied to sacroiliac joints have been estimated for dogs of varying weights evaluating only bony components (17). Authors of that report concluded that sacroiliac joint biomechanics were likely affected primarily by dog weight and the shapes of the bony components of the sacroiliac joints. However, soft tissue components were not evaluated. Finite element analysis (FEA) is the process of running numerous mathematical calculations on a computer-generated model that is broken down into simple geometry (mesh) (18). This model can then be used in a simulation to predict outcomes based off user-input. Previous human studies have demonstrated that FEA can model stress and strain in the SIJ and other pelvic structures (18–24). Computed tomography (CT) or magnetic resonance imaging (MRI) techniques were used in these studies to construct three-dimensional (3D) models based on patient-specific anatomy and conformation. Forces are applied to any specific spot on the extrapolated 3D model to calculate stresses and strains in different areas of that model. However, these previous studies did not describe repeatability of FEA measurements. Finite element analysis methods for modeling the canine lumbar spine (25) pelvis (26), and femur (27, 28) have been published. At the time of the current study, no peer-reviewed publications were found describing FEA methodologies for modeling canine SIJ ligament strain based on patient-specific CT data. Factors contributing to SIJ lesions such as acute injury and chronic disease in working dog populations are poorly understood. A computational SIJ model would allow for complex modeling of the associated bone, ligaments and muscles; and unconstrained motion and modulation of the inherent anatomical and structural features. The 3-D model could be subjected to physiological and injury-inducing external loads for scrutiny of response; therein revealing underlying structural and mechanical properties inherent to the SIJ bones while under dynamic or kinematic simulation and ligament, muscle, and motions loads.

The current, preliminary, two-part study explored two methodologies for possible future use by researchers interested in quantifying effects of working tasks on canine sacroiliac joints: numbers of SIJ CT lesions/dog and SIJ ligament strain calculated using FEA. The first part of the study tested the hypothesis that numbers of SIJ CT lesions/dog would differ among work status groups in a sample of young Labrador retriever working dogs. The second part of the study tested hypotheses that a methodology for conducting FEA of SIJ ligament strain in the static canine pelvis using patient-specific CT data would be feasible and that models based on this methodology would yield repeatable measures of SIJ ligament strain. Long term goals were to lend insight into dynamic physiologic, anatomic, kinematic, and 3-D mechanical conditions that have the potential to lead to working dog SIJ lesions. Findings could also play a role in future predictive modeling and identifying critical conditions and mechanisms influencing the proclivity of SIJ injury particularly in working dogs that may have otherwise gone undetected, or worse been a source of morbidity leading to abbreviated service.

## Methods

### Dogs

The study was a retrospective, secondary analysis, exploratory design. Labrador retriever working dogs, aged 11–48 months, that had undergone lumbosacral CT scanning for two, previous research studies were included (29, 30). Due to the retrospective nature of the study, institutional animal care, and use committee approval was not required. However, investigators in the previous research studies granted permission to retrieve and use the clinical and CT data for the current study. Scans had been acquired at two referral hospitals using 16-slice CT scanners and standardized protocols (Ryan Veterinary Hospital, PennVet, University of Pennsylvania, Brightspeed S, GE Medical systems, 0.625 mm slice thickness, body filter, bone convolution kernel; and the LTC Daniel E. Holland Memorial Military Working Dog Hospital, Lackland Joint Base, San Antonio, Lightspeed VCT, GE Medical Systems, 0.625 mm slice thickness, body filter, standard convolution kernel). All dogs had been placed in dorsal recumbency with the hips positioned in maximal flexion and maximal extension for scanning. To minimize outside sources of variation due to positioning, all interpretations and analyses for the current study were based on scans that had been acquired in the flexed hip position.

### Part 1: Comparisons Between Numbers of SIJ CT Lesions/Dog and Dog Work Status

A total of 22 dogs met the inclusion criteria and were classified into the following groups for analyses: sex (male, female), neuter status (intact, spayed, neutered), age (younger, 11–30 months; and older, 31–48 months), and work status at the time of CT scanning (Detection, Breeder, and Other). Dogs listed as “in-training detection” or “detection” in their records were classified into the Detection work status group. Intact female dogs with no described work status in their records were classified into the Breeder work-status group. Dogs that were not intact female dogs and that had no described work status were classified into the Other work status group. An undergraduate research student (KO) and graduate student (MC) made consensus decisions for dog group classifications and clinical data recording, without knowledge of CT findings.

The CT scans for included dogs were retrieved and reviewed by an ACVR-Board Certified Veterinary Radiologist (JJ), who was unaware of clinical findings or dog group status at the time of data recording (Horos Version 2.0.2 on MacOS Sierra Version 10.12.3 IMac by Apple Inc). Scans were interpreted in random order and the SIJ to be evaluated first (left or right) was determined by a coin toss. Numbers of the following CT lesions were recorded for each SIJ, based on criteria described in a previous publication (16): subchondral sclerosis, subchondral cysts, subchondral erosions, subarticular clefts, intra-articular ankylosis, and para-articular ankylosis. The radiologist used 3D multiplanar reformatting and adjusted window/level settings as needed for making decisions. Lesions had to be detected in at least two orthogonal planes before they were recorded as present. Additional observations at the time of interpretation were also recorded.

In consultation with a statistician (WB), the undergraduate research student (KO) selected and performed statistical comparisons of mean numbers of SIJ CT lesions/dog among dog groups. All statistical analyses were performed using commercially available statistics software (JMP Pro Statistical Software). Analysis of Variance (ANOVA) was performed to determine if there were significant differences in the mean number of SIJ lesions/dog among the work status groups. Assumptions for ANOVA were assessed and found to be satisfied. If evidence of difference among the group means was found, Fisher's Protected Least Significant Difference (LSD) Test was used to compare the three, work status group means. This method was chosen because this was an exploratory study; Fisher's Protected LSD is appropriate to reduce the chance of missing important differences to be examined in the future. Effects of sex and age on the work status group mean comparisons were evaluated by repeating the group comparison analysis with an ANOVA that included the main effects of sex and age, as well as the interactions of work status group with sex and age. Also because this was an exploratory study, any *p* < 0.10 was considered evidence of possible differences in the work groups.

### Part 2: Development of an FEA Method for Modeling Canine SIJ Ligament Strain Using CT Data and Repeatability of Ligament Strain Measurements

#### Design and Inclusion Criteria

The CT scans were further evaluated for a dog meeting the following criteria: (1) the CT scan had to include images of the complete pelvis and had to demonstrate minimal or no SIJ CT lesions with the intention to minimize possible disease-based effects on SIJ ligament strain values; (2) the CT scan had to include both ischiatic tuberosities so that all sacroiliac ligament attachments could be modeled; (3) the CT scans also had to include a bone algorithm study with 0.625 mm slice thickness such that boney edges could be clearly distinguished for segmentation (31). These decisions for subject selection were made by an ACVR-certified Veterinary Radiologist (JJ).

The dog selected for this part of the study was a 25 kg, 20-month-old, female, purebred Labrador retriever working dog. The pelvic CT scan DICOM files from this dog were exported to a personal computer (Lenovo ThinkPad S1 Yoga, Intel Core i5-4200U CPU 1.60 GHz, 8.00 GB, Hong Kong, China; Microsoft Windows 10, Redmond, WA) and a series of pilot studies were conducted to develop the FEA methods described below (32).

#### Segmentation Procedures

**Figure 1:**
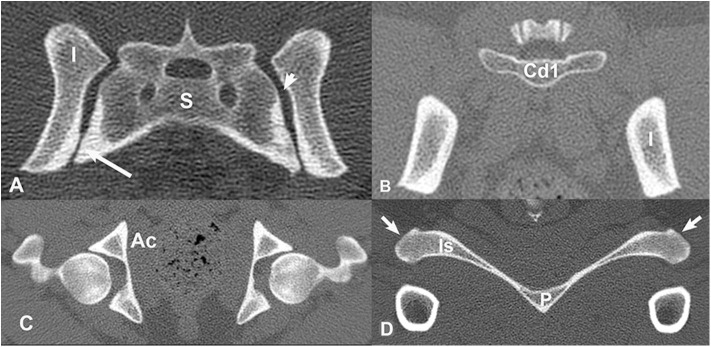
Transverse, bone window CT images illustrating pelvic bone structures that were segmented for construction of the three-dimensional model. **(A)** S, sacrum; I, ilium; Large arrow = synovial component of sacroiliac joint space. Small arrow = fibrous component of sacroiliac joint space. **(B)** Cd1 = first caudal vertebra, I, ilium; **(C)** A, acetabulum; **(D)** Is, ischium; P, pubis; arrows = ischiatic tuberosities. Images are displayed with dorsal at the top and the patient's left to the viewer's right.

Segmentation of the individual pelvic bones (sacrum, ilium, ischium, acetabulum, pubis, and first caudal vertebra) was done using transverse CT images (Figure 1) and a three-dimensional (3D) image analysis freeware program (3D Slicer, version 4.5.0, http://www.slicer.org). A semi-automated process using a tool called the “threshold effect” was first run throughout all the imported digital imaging and communications in medicine (DICOM) CT image files for segmentation of the majority of pelvic bones. A total of 226 CT images were used for the segmentation. Next, a “paint effect” tool was manually drawn on the remainder of bone not covered by the “threshold effect” tool. This was conducted on each image using the three different planes (transverse, sagittal, and dorsal). Each bone was segmented using a different color label for ease of manipulation. Special care was especially taken to manually trace margins in areas where the bones came in close contact with each other (the sacrum and ilia). Once the individual pelvic bones listed above were segmented, a surface model was created by exporting each bone segmentation as a stereolithography (.STL) file. Next, each STL surface model was individually imported into a mesh generation software (ICEM CFD 17.0) to create surface mesh based off the geometric .STL file. The surface mesh of each bone was set to roughly have the same size elements such that the small features of bones are reasonably modeled to the live specimen. The surface meshes were then exported to a .STL file once again.

To enable solid model operations of the bones (i.e., Boolean operations) of the bones, the surface mesh .STL file is then converted to an Initial Graphics Exchange Specification (.IGS) file using a 3D solid modeling tool (SolidWorks 2016, Waltham, MA). After the geometric model was in the correct file format, the engineering simulation software (ANSYS Workbench) was used for setting up the geometry, creating the ligaments and joints, generating the finite element mesh, assigning the material properties, and performing the finite element analysis.

#### Material Properties of Bone, Ligament, and Joints

The pelvic bones, sacroiliac joints, sacrocaudal joints, and sacroiliac joint ligaments were included in the model and all assumptions for the model were based on human pelvic tissue properties (21) (Table 1). The ligaments were modeled to resist only tension (i.e., there was no stiffness in compression).

**Table 1:** Isotropic elasticity properties that were used for modeling the pelvic bones in the current canine study.

**Youngs modulus (Pa)**	**Posson's ratio**	**Bulk modulus (Pa)**	**Shear modulus (Pa)**
1.7E+10	0.3	1.4167E+10	6.5385E+09

*These were previously published for human pelvic bones at room temperature (27°C) (21)*.

Ligaments in the pelvic region (dorsal sacroiliac ligament, ventral sacroiliac ligament, and sacrotuberous ligament) were modeled by using non-linear springs. Connection points of the ligaments on the bones were first identified based on a canine anatomy reference textbook (11). Then the non-linear springs were inserted to represent the ligaments. The non-linear tensile stiffness of each of the ligaments was defined as a piecewise linear strain-stiffness curve, using values listed in Table 2.

**Table 2:** Values of ligament stiffness as function of tensile strain that were used in the SIJ FEA model.

**Ligament strain**	**<2.5%**	**<5%**	**<10%**	**>10%**
**Stiffness (N/mm)**	39	55	103	100

**Figure 2:**
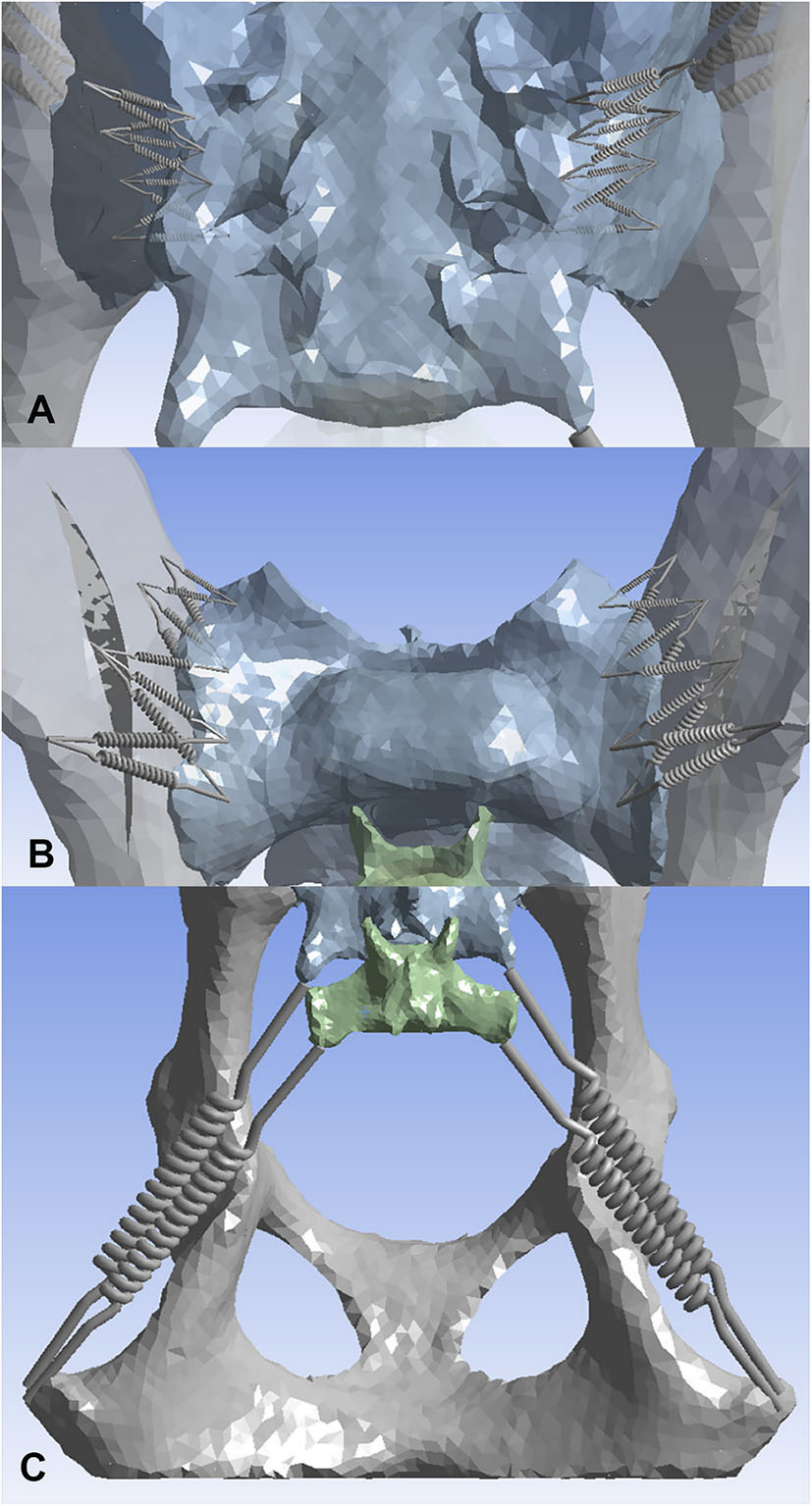
Three-dimensional models displaying non-linear springs that were used to represent the sacroiliac joint ligaments. **(A)** dorsal sacroiliac ligaments, **(B)** ventral sacroiliac ligaments, **(C)** sacrotuberous ligament. Ten non-linear springs per side were used on both the dorsal sacroiliac ligament and the ventral sacroiliac ligament. Four points of interests were first picked on the sacrum and the ilium of both sides, followed by connecting the non-linear springs in a crisscross pattern. This pattern was used to mimic more of the ligaments sheet-like properties. For the sacrotuberous ligament, two non-linear springs per side were used. The ligament attachment sites were placed on the margins of the transverse processes of the third sacral and first caudal vertebrae, and the ischiatic tuberosities.

**Figure 3:**
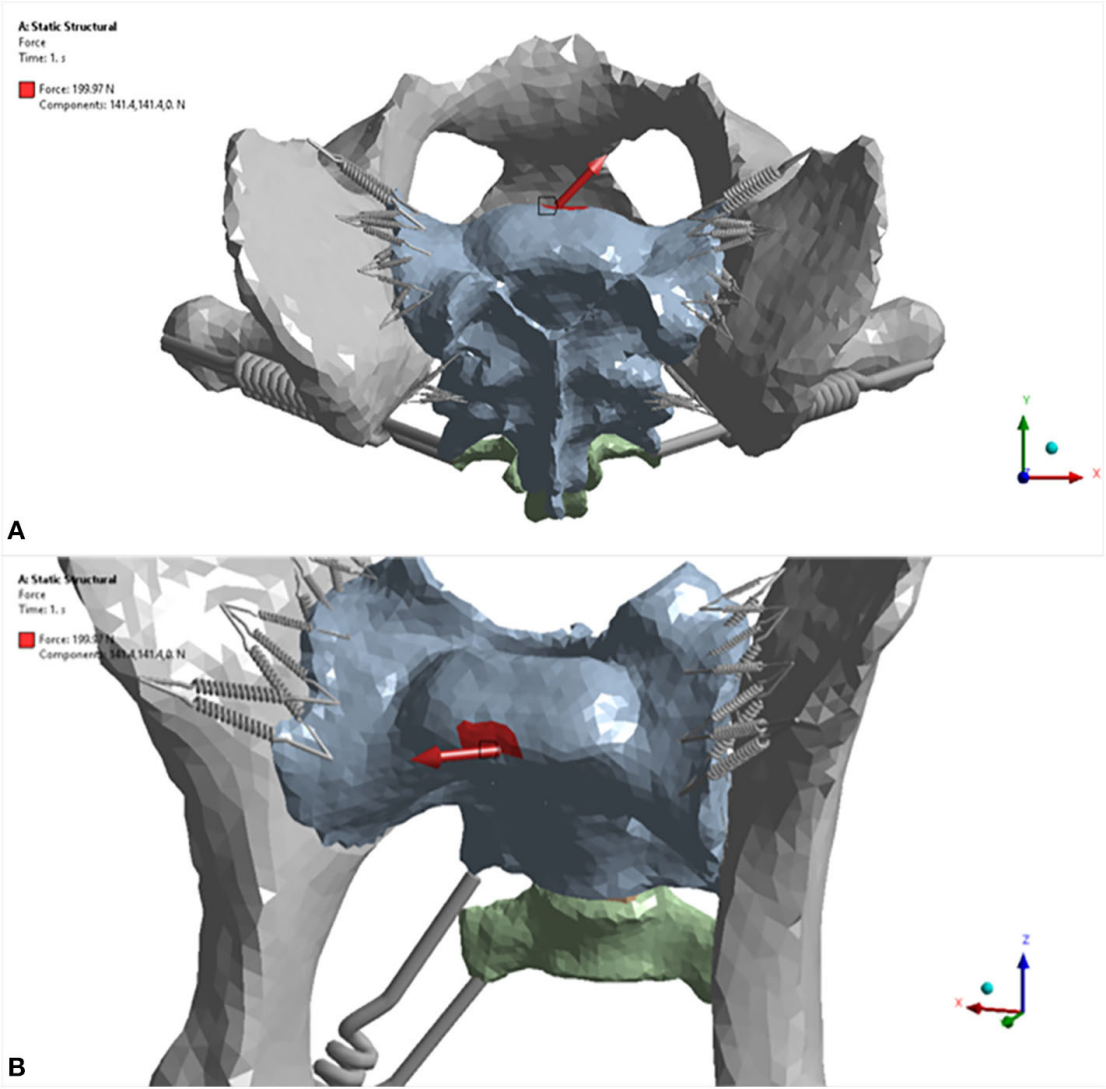
Three-dimensional models displaying load scenario No. 5 for the dog pelvis (red arrow). **(A)** craniodorsal view; **(B)** cranioventral view. For this load scenario, 141.4 N was placed in the X direction and 141.4 N in the Y direction. The model was fixed in place at the level of the acetabulum.

Ten non-linear springs were placed on each side (L, R) for the dorsal and ventral sacroiliac ligaments (Figure 2). To limit sliding motion and provide more of a sheet-like property, a crisscross pattern was developed. The 10 non-linear springs on each side for each ligament were all connected via a crisscross as shown in the figure. The sacrotuberous ligament was demonstrated using two non-linear springs and a “Y” configuration (Figure 3).

To define its non-linear tensile stiffness and zero compression stiffness, the sacroiliac joint was modeled using 10 non-linear springs per side. Each of the non-linear springs share the same properties as the non-linear springs that were used in the ligaments. Next, the joint between the sacrum and the first caudal vertebrae was modeled (sacrocaudal joint). The modulus of elasticity E was defined using the equation below

(1) $$\begin{array}{r} E=\{\begin{array}{cc} 0 & for\;\varepsilon \geq 0\\ 20.71\varepsilon +234\varepsilon^{2} & for\;\varepsilon <0 \end{array} \end{array}$$

where ε is the strain (21). The above equation gives an increasing stiffness when a joint is compressed and a zero stiffness when it is stretched. This implies that the joint does not resist force separating the bones to which it is connected. Equation (1) can be rewritten in the form of stress-strain relation:

(2) $$\begin{array}{r} \sigma =\{\begin{array}{cc} 0 & for\;\varepsilon \geq 0\\ 10.355\varepsilon^{2}+78\varepsilon^{3} & for\;\varepsilon <0 \end{array} \end{array}$$

where σ is the normal stress with unit of MPa. In this work, the non-linear compressive stress-strain behavior of the joint is modeled as a hyperelastic material response. The 3-paramater Mooney-Rivlin model is employed and the model parameters are calculated by fitting the stress-strain curve defined by Equation (2). The solid model of the joint was constructed by manually creating a joint volume between the C1 vertebrae and the sacrum through a combination of drawing, extrusion, and Boolean operations.

#### Finite Element Analysis Settings and Loading Conditions

**Figure 4:**
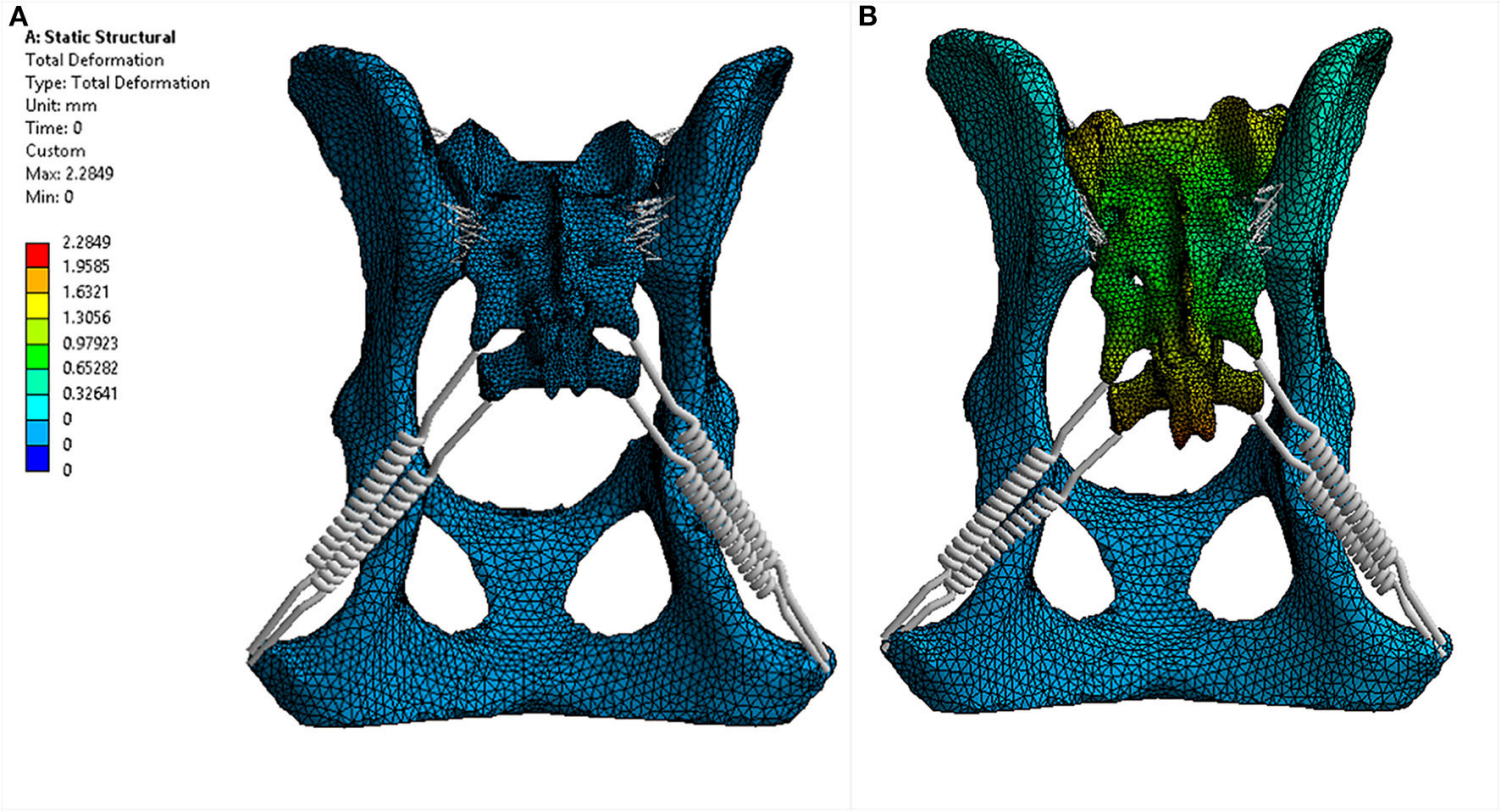
Deformation of the proposed canine pelvis model due to a 200 N force applied to the sacrum (load scenario 7). **(A)** dorsal 3D view of the pelvis prior to applied force. **(B)** dorsal 3D view of the pelvis with the applied force (141.4 N in the X direction and −141.4 N in the Y direction).

**Figure 5:**
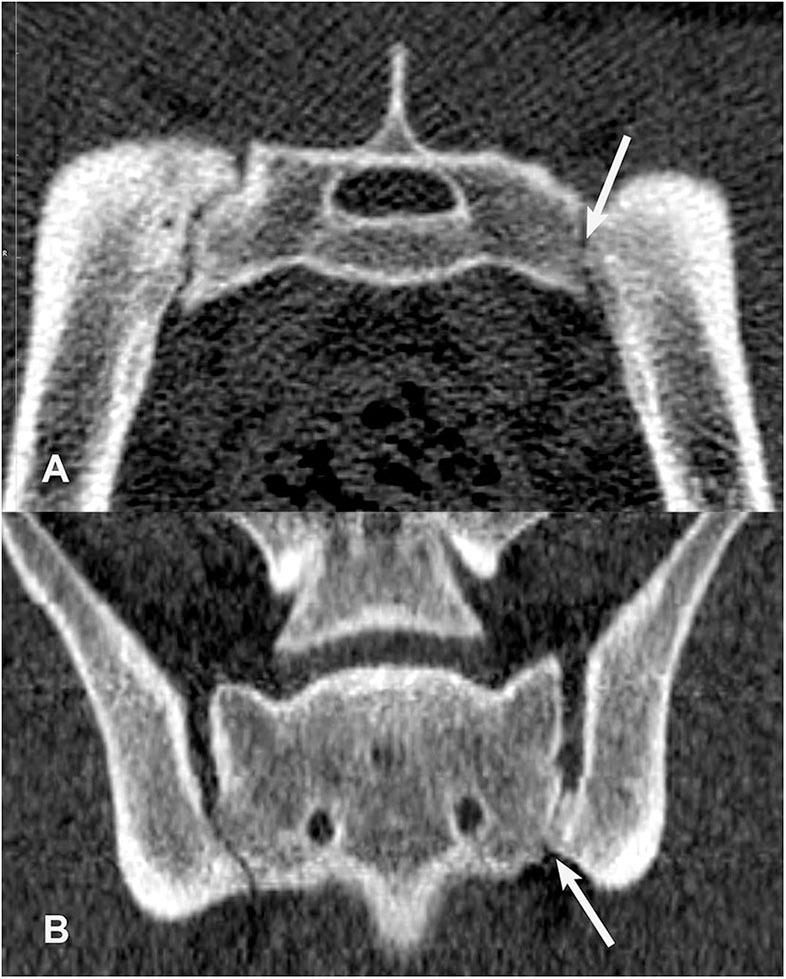
Transverse **(A)** and dorsal planar **(B)** CT images illustrating an intra-articular ankylosis lesion in the left sacroiliac joint (arrows). The left and right sacroiliac joints also appear asymmetrical in size and shape. The transverse images are displayed with dorsal at the top and the patient's left to the viewer's right. Dorsal planar images are displayed with cranial at the top and the patient's left to the viewer's right.

For each trial, the model was fixed in space with a fixed support applied to each side of the acetabulum. Eight different force loads (scenarios) were applied to the sacrum as shown in Table 3. The location where the forces were applied is illustrated in Figure 4. Strain (elongation per unit length) in the ligaments caused by the loads was calculated and recorded for each ligament (non-linear spring) in each scenario. For each scenario, the strains of all non-linear springs in each ligament group on each side were averaged. The results obtained from the five trials were then compared and analyzed statistically. Deformation and equivalent stresses were also obtained from the analysis (Figure 5). The deformed position of the dog pelvis under the load scenario 7 is shown in Figure 5B. For comparison, the unreformed pelvis is also shown on the right. Notice that the displacement in the plot is exaggerated to demonstrate the deformation more clearly.

**Table 3:** Eight loading scenarios that were applied to the sacrum in the model.

**Scenario**	**Force in x-direction (*N*)**	**Force in y-direction (*N*)**
1	0	200
2	0	−200
3	200	0
4	−200	0
5	141.4	141.4
6	−141.4	−141.4
7	141.4	−141.4
8	−141.4	141.4

#### Repeatability of Ligament Strain Measurements Using the FEA Method

Upon completion of the pathway from segmentation to model development to finite element analysis, five trials separated by exactly 1 week apart were conducted by a single observer (MC). Strain values of ligaments were recorded for each trial using different loading scenarios. The trials consisted of the steps discussed above and involved four software programs: a three-dimensional (3D) image analysis program (3D Slicer, version 4.5.0, http://www.slicer.org), mesh generation software (ICEM CFD 17.0, ANSYS, Canonsburg, PA), 3D computer-aided design program (SolidWorks 2016, Waltham, MA), and engineering simulation software (ANSYS Workbench 17.0, Canonsburg, PA). Software used was either free of charge or was provided under Clemson University's licensing. All trials were performed using one workstation (Lenovo ThinkPad S1 Yoga, Intel Core i5-4200U CPU 1.60 GHz, 8.00 GB, Hong Kong, China; Microsoft Windows 10, Redmond, WA). Finite element analysis was conducted on a different workstation (Apple MacBook Pro 17, 2.4 GHz quad-core Intel Core i7, 16.00 GB, Cupertino, California; Microsoft Windows 7, Redmond, WA).

Statistical analyses were selected and conducted in consultation with a statistician (JS). Strain values (y-axis) by side and ligament group, vs. trial (x-axis) were examined visually using commercially available statistics software (JMP, Version 14.0, SAS Institute Inc., Cary, NC, 1989-2007). A regression analysis was used to compare the strain value of each ligament (dependent variable) to determine if the values were similar throughout the five trials (independent variable). A total of 48 regression analyses were run (8 force load scenarios x 3 ligaments x 2 sides). Our null hypothesis was that the methods are repeatable (slope of the regression line is zero) while the alternative hypothesis was methods are not repeatable (slope of the regression line is not zero). Multiple comparison adjustments were not employed for this exploratory analysis.

## Results

### Part 1: Comparisons Between Numbers of SIJ CT Lesions/Dog and Dog Work Status

**Figure 6:**
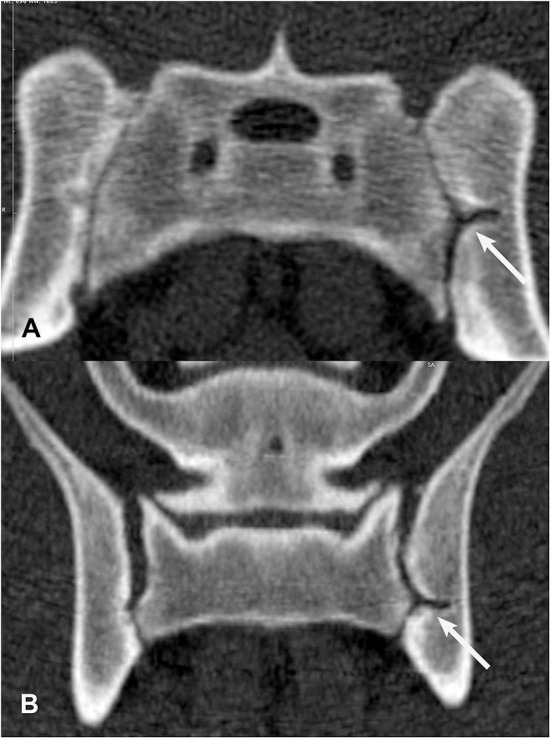
Transverse **(A)** and dorsal planar **(B)** CT images illustrating a subarticular cleft lesion in the left sacroiliac joint (arrows). Subchondral erosion lesions are also evident in the right sacroiliac joint. The transverse images are displayed with dorsal at the top and the patient's left to the viewer's right. Dorsal planar images are displayed with cranial at the top and the patient's left to the viewer's right.

**Figure 7:**
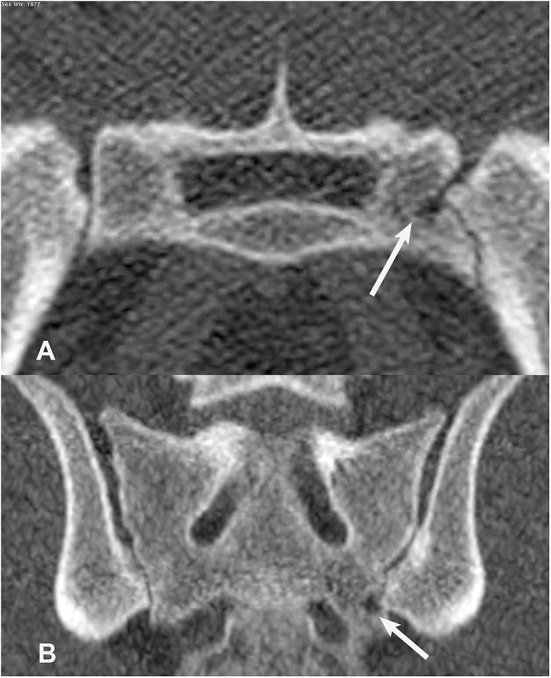
Transverse **(A)** and dorsal planar **(B)** CT images illustrating a subchondral cyst lesion in the left sacroiliac joint (arrows). A subchondral sclerosis lesion is also evident in the dorsal planar view of the left sacroiliac joint. The transverse images are displayed with dorsal at the top and the patient's left to the viewer's right. Dorsal planar images are displayed with cranial at the top and the patient's left to the viewer's right.

**Figure 8:**
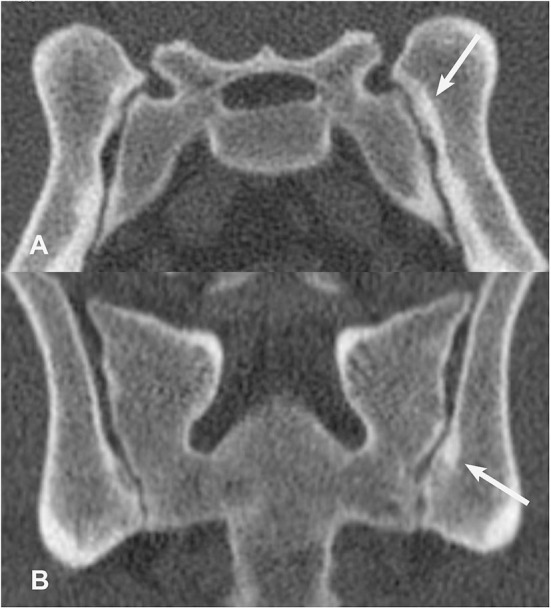
Transverse **(A)** and dorsal planar **(B)** CT images illustrating a subchondral sclerosis lesion in the left sacroiliac joint (arrows). A subchondral erosion lesion is also evident in the dorsal planar view of the right sacroiliac joint. The transverse images are displayed with dorsal at the top and the patient's left to the viewer's right. Dorsal planar images are displayed with cranial at the top and the patient's left to the viewer's right.

**Figure 9:**
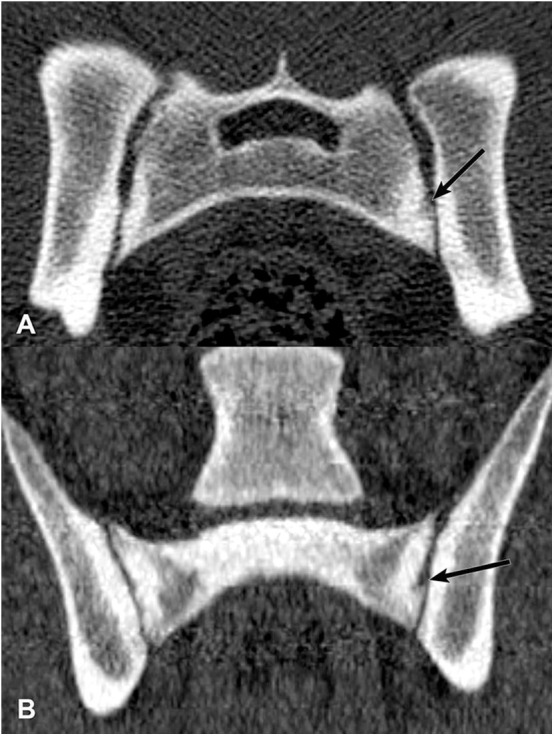
Transverse **(A)** and dorsal planar **(B)** CT images illustrating a subchondral erosion lesion in the left sacroiliac joint (arrows). A subchondral sclerosis lesion is also evident surrounding the erosion. The transverse images are displayed with dorsal at the top and the patient's left to the viewer's right. Dorsal planar images are displayed with cranial at the top and the patient's left to the viewer's right.

**Figure 10:**
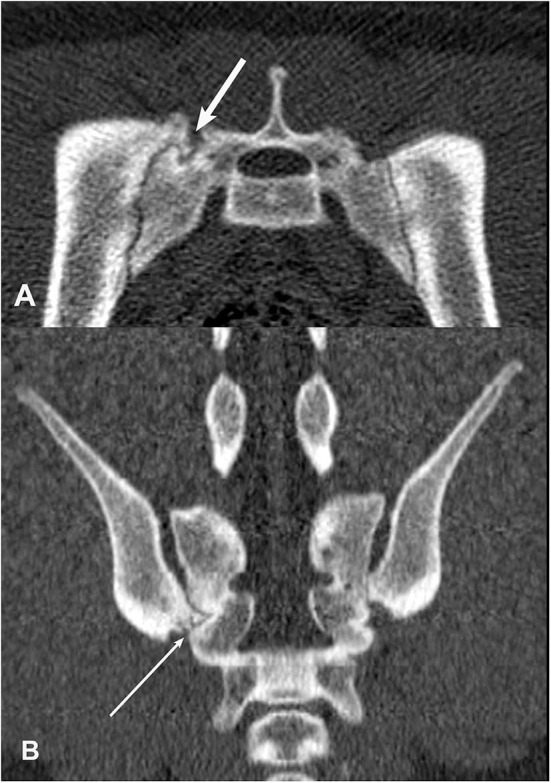
Transverse **(A)** and dorsal planar **(B)** CT images illustrating an intra-articular bone spur lesion in the right sacroiliac joint (arrows). The left and right sacroiliac joints are asymmetrical in shape and size. Subchondral erosion lesions are visible in both joints in the transverse view. The transverse images are displayed with dorsal at the top and the patient's left to the viewer's right. Dorsal planar images are displayed with cranial at the top and the patient's left to the viewer's right.

Of the 22 included dogs, four were in training for future police or search and rescue work and 18 were military working dogs. Ten dogs were categorized as Detection, 6 dogs categorized as Breeder, and 6 dogs categorized as Other (Table 4). Thirteen dogs were classified into the younger age group (age 11–30 months) and nine dogs were classified into the older age group (age 31–48 months). Of the younger age group dogs, nine were intact males, and four were intact females. Within the younger age group, there were six Detection dogs (four males and two females), two Breeders, and five Others (all male). The older age group was composed of six females and three males. There was one neutered male and one spayed female and the rest of the dogs were intact. There were four Detection dogs (one intact male, one neutered male, one intact female, one spayed female), four Breeder dogs, and one Other dog (intact female). While neuter status may have implications for joint and ligament health, its effect could not be assessed in the present study due to the small number of neutered/spayed animals in this sample (33, 34). In addition to detecting SIJ CT lesions that have been previously described (16) (Figures 6–9), the veterinary radiologist detected a new type of CT SIJ lesion and termed it “intra-articular bone spur” (Figure 10). None of the dogs were found to have para-articular ankylosis lesions.

**Table 4:** Descriptive summary of clinical and computed tomography findings for the 22 Labrador retriever working dogs included in the sample.

**Work status category**	**Number of dogs**	**Age range (months)**	**Weight range (kg)**	**Sex and neuter status**	**Average # SIJ CT lesions/dog in each lesion category**						
					**SCy**	**SE**	**SS**	**PAA**	**IAA**	**SCl**	**IBS**
Breeder	6	28–48	22.6–27.2	6 FI	0.42	1.58	1.17	0	1.33	0.5	0.5
Detection	10	11–41	26–34	2 FI 1 FS MI 1 MN	0.85	1.2	0.4	0	0.7	1.1	0.45
Other	6	14-32	19.1–29	1 FI 5 MI	1.58	1.75	0.67	0	0.58	1.17	1.08

*kg, kilogram; CT, computed tomography; FI, female intact; FS, female spayed; MI, male intact; MN, male neutered; SCy, subchondral cyst; SE, subchondral erosion; SS, subchondral sclerosis; PAA, para-articular ankylosis; IAA, intra-articular ankylosis; SCl, subarticular cleft; IBS, intra-articular bone spur*.

The ANOVA testing identified no significant difference in the mean number of SIJ CT lesions/dog across work status categories for subchondral erosions, subarticular clefts, intra-articular bone spurs, or intra-articular ankylosis lesions for the right SIJ, left SIJ, or in the total joint with both sides added together. Analysis of the total number of SIJ CT lesions found in each work status group yielded no significant difference in the means among the three categories.

No significant differences were found in the occurrences of subchondral sclerosis and subchondral cyst lesions among the work status groups. There was evidence that the mean number of subchondral sclerosis lesions in the left SIJ differed among work status groups (*p* = 0.0780). Specifically, there appeared to be a greater number of subchondral sclerosis lesions identified in the left SIJ of Breeders than of Detection dogs (p_adj_ = 0.0258). There was not evidence of a difference between Other dogs and Breeders or Detection dogs in mean number of subchondral sclerosis lesions in the left SIJ. There was not evidence of a difference in the mean number of subchondral sclerosis lesions in the right SIJ among work status categories. The mean number of subchondral cysts in the right SIJ was different among the work status categories (*p* = 0.0296). The mean number of subchondral cysts in the right joints of Breeder dogs was significantly lower than for Other dogs (p_adj_ = 0.0227/2) and for Detection dogs (p_adj_ = 0.0154/2). There was not evidence of a difference in mean number of subchondral cysts between Other dogs and Detection dogs. These results were isolated to the right SIJ as no significant difference was seen among the work status categories for the mean number of subchondral cysts in the left side of the joint.

When introducing the variables of age and sex to the mean number of the subchondral sclerosis lesions seen in the left side of the SIJ across work status groups, female dogs in the “older” (31–48 months) group appear slightly different. Dogs in the Other work status group had a subjectively greater number of these lesions, followed by Breeders, while subjectively fewer lesions of this type were seen for Detection dogs.

When the same variables were introduced for the mean number of subchondral cysts in the right SIJ across work status groups, similar results were seen. Female dogs in age group 2 (i.e., older) working as Detection dogs and dogs categorized as Other showed a higher mean number of subchondral cysts in the right SIJ than Breeder females in the same age group.

### Development of FEA Method for Modeling Canine Sacroiliac Joint Ligament Strain Using CT Data and Repeatability of Ligament Strain Measurements

Results of the 48 hypothesis tests for the selected dog are summarized in Table 5. We failed to reject the null hypothesis that the method is repeatable in approximately 98% of the tests at each combination of strain, ligament, and side (47 out of 48). We rejected the null hypothesis that the method is not repeatable in approximately 2% of the tests at each combination of strain, ligament, and side (1 out of 48). The hypothesis test that was not statistically repeatable was in force load (scenario) 8 for the left ventral sacroiliac ligament (*P* = 0.0175). Strain values under this scenario for this ligament ranged from −0.02343 (trial 1) to −0.01422 (trial 5).

**Table 5:** Regression analysis results [slope estimate (95% confidence interval limits); *p*-value] for sacroiliac ligament strain values (dependent variable) vs. trial (independent variable) for each ligament (Dorsal Sacroiliac, Sacrotuberous, and Ventral Sacroiliac) on each side (L, R) for each of 8 scenarios.

**Ligament**	**Scenario number**	**Slope estimate (95% confidence interval limits);** ***p*****-value for SIJ ligament strain values (dependent variable)**	
		**Right side**	**Left side**
Dorsal sacroiliac	1	−0.001 (−0.004, 0.003); 0.6841	0 (−0.002, 0.001); 0.8539
	2	−0.002 (−0.007, 0.003); 0.3029	0.006 (0, 0.011); 0.058
	3	−0.002 (−0.007, 0.004); 0.4442	0.006 (0, 0.011); 0.0511
	4	−0.001 (−0.009, 0.007); 0.6946	0.001 (−0.001, 0.003); 0.3748
	5	0 (−0.006, 0.005); 0.809	−0.002 (−0.004, 0.001); 0.1356
	6	−0.002 (−0.009, 0.005); 0.4267	0.002 (−0.002, 0.005); 0.2197
	7	−0.002 (−0.006, 0.003); 0.3843	0.006 (−0.001, 0.014); 0.0689
	8	0.001 (−0.002, 0.004); 0.2746	0 (−0.001, 0.001); 0.7566
Sacrotuberous	1	0.001 (−0.001, 0.002); 0.1631	0.001 (−0.001, 0.003); 0.2797
	2	0.001 (−0.004, 0.005); 0.572	0.001 (−0.003, 0.005); 0.6195
	3	−0.001 (−0.003, 0.002); 0.5198	0 (−0.003, 0.002); 0.5664
	4	0.002 (−0.001, 0.005); 0.1488	0.002 (−0.001, 0.005); 0.1347
	5	0 (−0.004, 0.005); 0.8486	0 (−0.004, 0.005); 0.7552
	6	0.002 (−0.003, 0.008); 0.2248	0.002 (−0.002, 0.007); 0.1914
	7	−0.001 (−0.004, 0.002); 0.4739	−0.001 (−0.004, 0.002); 0.4105
	8	0.001 (0, 0.001); 0.138	0.001 (−0.001, 0.002); 0.2084
Ventral sacroiliac	1	−0.001 (−0.005, 0.003); 0.5834	0 (−0.003, 0.003); 0.8687
	2	0 (−0.007, 0.007); 0.9036	0.004 (−0.01, 0.018); 0.396
	3	−0.002 (−0.008, 0.003); 0.2856	0.004 (−0.006, 0.015); 0.2922
	4	0.002 (−0.008, 0.011); 0.602	−0.002 (−0.007, 0.004); 0.4572
	5	−0.001 (−0.005, 0.003); 0.3987	−0.003 (−0.012, 0.007); 0.4644
	6	0.002 (−0.007, 0.011); 0.5244	0 (−0.009, 0.008); 0.8955
	7	−0.002 (−0.008, 0.004); 0.2908	0.004 (−0.004, 0.013); 0.1929
	8	0.002 (0.001, 0.004); ^*^0.0175	−0.001 (−0.002, 0.001); 0.286

* *and bold font indicates statistically significant difference; “Scenario” was defined as the different loading conditions (different forces placed on different axes). “Trial” was the entire process (segmentation, model creation, analysis including all 8 scenarios) each separated by a week*.

## Discussion

The intentions of this two-part, preliminary study were to introduce two quantitative CT methods; with the long term goal of supporting future research studies characterizing effects of working tasks in SIJ. We explored application of these two methods in a small sample of young, Labrador retriever working dogs. We described a preliminary assessment of the presence of SIJ CT lesions in young working dog groups. We also introduced a new SIJ lesion, termed “intra-articular bone spur.” Although no significant differences were found for the mean number of SIJ lesions/dog, significant differences in subchondral sclerosis and subchondral cyst lesions were observed among work status groups when comparing individual sides of the joints.

### Part 1: Comparisons Between Numbers of SIJ CT Lesions/Dog and Dog Work Status

Findings from the first part of the study did not support the hypothesis that mean numbers of SIJ CT lesions/dog would differ among work status groups. However, when SIJ side (right or left) was examined, significant differences among work status groups were found for subchondral sclerosis and subchondral cyst lesions. Breeders were found to have a higher number of subchondral sclerosis lesions in the left SIJ when compared to that of Detection dogs while both Detection and Other dogs had higher numbers of subchondral cysts found in the right SIJ when each was compared to Breeder dogs. Age and sex also appeared to play a role in the number of lesions found in the SIJ with older (31–48 months) females having a greater number of both subchondral sclerosis and subchondral cyst lesions identified across all three categories when comparing the individual sides of the joints. These preliminary results suggest that multiple factors such as work status, age, and sex may contribute to the development of SIJ lesions in working dogs.

A previously unreported SIJ CT lesion was observed in some of the dogs and authors introduced the term “intra-articular bone spur.” These bone spurs were considered to most likely be enthesiophytes that formed at the interosseous ligament attachment sites. Though not histopathologically confirmed in the dogs of the current study, there is histologic evidence of a transitional zone of fibrocartilage extending into the ligamentous portion of the sacroiliac joint (12). This zone can develop degenerative changes in dogs as young as 5 months of age, with histologic evidence of cartilage matrix splitting and chondrocyte proliferation at ligamentous attachment sites. In people, when placed under unusual tensile strain, ligamentous entheses can undergo degenerative changes that make them more likely to tear outright, pulling away a portion of the underlying subchondral bone resulting in enthesiophytes (35, 36). The presence of these intra-articular enthesiophytes in young Labrador retriever working dogs could be an indication of cumulative mechanical energy (or overuse injury); or indicators that intense, repetitive training techniques employed in puppies employed in puppies in preparation for various lines of duty could possibly be detrimentally placing strain on the joint and its ligaments (6).

In this study, Breeders were found to have a higher mean number of subchondral sclerosis lesions in the left SIJ when compared to that of Detection dogs. This was an unexpected finding because unspayed, female dogs are not typically trained or used for working tasks. Possible theories could be that dogs voluntarily performed repetitive upright postures in their kennels or that whelping and hormonal influences could have altered SIJ ligament rigidity. In people, changes in the pubic symphysis of women caused by degeneration or vertical displacement was positively correlated with SIJ pain, despite being not significantly associated with parous status (37). Estrogens and relaxins released in dogs during pregnancy have been shown influence the tensile properties of connective tissues by increasing the flexibility of both the pubic symphysis as well as the SIJ (38). The aforementioned puts the hip joints at a greater risk for instability and in turn affect the stability of the SIJ. Hormonally influenced ligament laxity combined with structural changes in the pubic bone and SIJ resulting from pregnancy, repeated estrus cycles in whelping intact females may contribute to SIJ instability. Based on Wolff's law, this could in turn predispose the subchondral bone to microfractures and subsequent sclerosis (39). Parity may also play a role in the degree of change within the SIJ, which may in turn influence the number of lesions seen, but further studies would be necessary to determine whether the number of litters a female carries is related to increased joint laxity when compared to females that have had fewer litters.

In the current study and in a previous canine cadaver study, subchondral cyst CT lesions appeared as discrete oval radiolucencies with surrounding sclerotic rims in the subchondral bone of the articular components of the SIJ, and subchondral sclerosis lesions appeared as focal areas of increased subchondral bone radioopacity (16). A previous canine study described histologic evidence of articular cartilage damage (including splitting of the cartilage matrix, proliferation of chondrocytes, and decreased glycosaminoglycan production in the cartilage matrix) in the synovial component of SIJ in dogs as early as 5 months of age (12). A “synovial lined recess” was identified in a 3-year-old dog. In the present study, there was a pattern for increased numbers of subchondral sclerosis and subchondral cyst lesions/dog in female dogs of the “older” (31–48 months) category. While not statistically significant, these differences could be preliminary evidence used to generate hypotheses in other studies that age and female sex could also be risk factors for injury or progression of microtraumas in the SIJ. All three work status categories were represented in this trend, with some variation in the specific frequencies amongst each category. There was significant lateralization in the mean numbers of subchondral sclerosis and subchondral cyst lesions/dog, with significantly more lesions/dog identified in one side of the joint but not the other. Breeder dogs were found to have a significantly higher occurrence of subchondral sclerosis lesions when the left side of the SIJ was compared to the left SIJ of detection dogs but no significant differences were found when comparing the number of lesions in the right SIJ or the mean number of lesions in the total joint of both groups. Additionally both Other and Detection dogs had a significantly higher number of subchondral cysts when comparing the right SIJ between Other vs. Breeder and Detection vs. Breeder but similarly, no significant differences were found between the pairings when comparing the left SIJ or the mean number of lesions in the total joint. Though possibly limited due to a lack of power, one theory for these observed side differences could be that dog handlers have a side preference when asking dogs to perform repetitive training and working tasks.

### Part 2: Development of the FEA Method for Modeling the Sacroiliac Joint and Ligaments Using CT Data and Repeatability of Ligament Strain Measurements

We developed a methodology for constructing a computer-based model of the canine pelvis using CT images and four computer software applications; one of which was freely available, while the other three required commercial licenses. Our methodology was adapted from a previously published, CT-derived, human pelvis FEA model (21). In-depth details on the process of creating the model were not available in the previous publication and no repeatability testing was described. To evaluate intra-observer repeatability in the current study, we determined that quantifiable parameters were needed for each trial and case number. The decision to average all of the strain values for each of the non-linear springs to obtain an average SIJ ligament strain value was developed for this reason. Intra-observer repeatability of this methodology was then tested by using regression analyses on the SIJ ligament strain values of five models separated by a week apart and performing FEA on each of the models. All models were given the same bone and ligament properties as those used in the previous human study and eight different loading conditions were placed on each model. Findings from the current study indicated that our procedure is repeatable (47 out of 48 load scenarios). The one variable that was not repeatable between trials was in the left ventral sacroiliac ligament under scenario 8 (−141.4 N placed on the sacrum base in the X direction and 141.4 N in the Y direction). It is possible that this non-repeatable variable occurred because of operator-based variability and non-physiological loading conditions.

After we had initiated the current study, a thesis was published that also described development of a biomechanical model using CT scans of a canine pelvis (40). Similar to ours, the study modeled bone and ligament properties for the canine pelvis based on the human literature. The study also included assumptions based on previously published tissue properties of canine long bones (41). Ligament placements were based on a veterinary textbook (11). Muscles and their properties were incorporated into the model using a cadaveric model and a separate biomechanical test for model validation. However, strain values for the sacroiliac ligaments and repeatability testing were not performed. In another study, a CT-derived canine FEA determined the presence of micro-motion following the repair of sacroiliac separation by way of different surgical fixations (26). These authors used a simplified model of the canine pelvis and, similar to the current study, modeled the bones as isotropic and elastic. Authors noted that the loading conditions they used for magnitude and directionality in their model were not physiologically realistic to the dog. The current study shared a similar trait to the previous study in that a large value in magnitude was used to capture the micromovements during FEA.

We considered repeatability testing to be important to assess the FEA methodology because operator decision-making occurs during multiple steps of the process. We chose to perform each of the trials at exactly 1 week apart in order to minimize effects of retained operator muscle memory on repeatability results. The segmentation portion of the process had the largest potential for operator-based variability due to the large number of CT slices and hand-traced bone regions of interest. Segmentation was also the most time-consuming stage of the procedure. The segmentation was performed using a freeware program (3D Slicer), and some automated thresholding was possible. However, this was otherwise mostly a time-consuming manual process. The use of newer software products that allow more automated thresholding could possibly reduce the time required and increase the accuracy of the method in the future. Non-linear spring placement to provide ligaments and joints in the model was also another source of variability. Not only the length and location of these springs, but also the axial direction, were possible sources of variability for the method. The sacroiliac joint has very complex anatomy, therefore finding the same location for placement of the springs on each trial was a particular challenge. Another possible source of variability was the exact positioning of the boundary conditions applied to the model. This included the location where the load was placed and where the model was held in space.

### Limitations and Conclusions

Several limitations need to be acknowledged for this preliminary, exploratory study. The sample sizes were small and these reduced the power of comparisons for work status, age, sex, and neuter status groups. The study sampled only one working dog breed, and therefore generalizability of findings for working dogs of other breeds remains unknown. The working tasks for dogs at these particular training centers may not be the same as those used at other training centers. The calculated ligament strain values for the single dog selected for Part 2 of the study were not validated based on actual bone and ligament properties in Labrador retrievers and were instead based on tissue properties published for humans. A previous review article described multiple limitations for using human tissue properties in canine musculoskeletal modeling (42). Muscles of the pelvis were not included in our model because we had intended to focus on adapting our methods to those described in a previous paper that was focused on FEA modeling of human SIJ ligament mechanical properties (21). A single dog was used for the 2nd part of the study and therefore effects of size, sex, breed, and age on ligament strain values were not tested. A single operator performed all trials and therefore inter-operator reliability of the technique was not tested. Histopathologic confirmations of the SIJ lesions and interosseous ligaments was not performed. This would have been unethethical in this otherwise healthy population of dogs. Finally, the FEA model was not validated with a cadaveric study.

In conclusion, this preliminary study introduced two quantitative CT measures for possible use in future research studies on effects of training and working tasks on SIJ in young working dogs. The first methodology was the number of SIJ CT lesions/dog and application of this method was explored using work status group comparisons in a small sample of young, Labrador retriever working dogs. The second methodology was SIJ ligament strain modeling based on patient-specific CT data and FEA. Intra-observer repeatability of SIJ ligament strain measurements using these models was tested in one dog and found to be very good. Further research is needed to examine specific training and working tasks required of young working dogs across multiple lines of duty and compare these using quantitative SIJ CT measures. Future studies are also needed to improve the SIJ ligament strain FEA model. A validation study should be performed using actual bone, joint, and muscle properties from dogs of representative breeds. The long-term goals for these research efforts would be to develop more evidence-based strategies for minimizing early retirement and maximizing quality of life for working dogs.

## Data Availability Statement

The datasets for this article are not publicly available because of hospital patient data confidentiality requirements. Requests to access military working dog datasets should be directed to Army Public Health Center ATTN: MCHB-IP-V 8252 Blackhawk Rd. Aberdeen Proving Ground, MD 21010-5403; email: usarmy.apg.medcom-aphc.mbx.iph-vet@mail.mil.

## Ethics Statement

The original, prospective military working dog study was reviewed and approved by LTC Daniel E. Holland Memorial Military Working Dog Hospital, Lackland Joint Base, TX; Institutional Animal Care and Use Committee # 2012–06; approval Aug. 12, 2012.

## Author Contributions

MC, JJ, GL, and JS: conception and design. MC, JJ, KO, and GL: acquisition of data. MC, JJ, GL, JS, KO, and WB: analysis and interpretation of data, revising article for intellectual content, and final approval of the completed article. MC, JJ, and KO: drafting the article. All authors contributed to the article and approved the submitted version.

## Conflict of Interest

The authors declare that the research was conducted in the absence of any commercial or financial relationships that could be construed as a potential conflict of interest.
